# Depth of field measures in pseudophakic eyes implanted with different type of presbyopia-correcting IOLS

**DOI:** 10.1038/s41598-021-91654-w

**Published:** 2021-06-08

**Authors:** Carlos Palomino-Bautista, Rubén Sánchez-Jean, David Carmona-Gonzalez, David P. Piñero, Ainhoa Molina-Martín

**Affiliations:** 1 Department of Ophthalmology, University Hospital Quirónsalud, Madrid, Spain; 2 Department of Optics, Pharmacology and Anatomy, University of Alicante, Crta San Vicente del Raspeig s/n, 03690 San Vicente del Raspeig, Alicante Spain; 3 Department of Ophthalmology, Vithas Medimar International Hospital, Alicante, Spain

**Keywords:** Outcomes research, Geriatrics

## Abstract

To evaluate depth of field (DOF) provided by different presbyopia-correcting intraocular lens (IOL) designs, comparing the results obtained using different criteria for defining the defocus tolerance. A total of 150 eyes undergoing cataract surgery were enrolled and divided into 6 groups depending on the IOL implanted: AT.LISA Tri (Carl Zeiss Meditec), FineVision (PhysIOL), PanOptix (Alcon Laboratories), Tecnis Symfony (Johnson & Johnson Vision), Miniwell (SIFI MedTech) and Tecnis Synergy (Johnson & Johnson Vision). Subjective DOF was obtained from defocus curves with absolute and relative criteria of tolerance of 0.1 logMAR. Aberrometry was also measured and the visual strehl optical transference function (VSOTF) with percentage of degradation of 90%, 80% and 60% was used to quantify objectively the DOF. Tecnis Symfony, Tecnis Synergy and Panoptix IOL groups showed better subjective and objective DOF compared to the rest of IOL groups, being these differences statistically significant differences (*p* < 0.001). Comparison between subjective and objective DOF showed that subjective measures were higher for all IOLs, being also these differences statistically significant for all groups (*p* < 0.001). A moderate significant correlation was found between absolute subjective criteria and VSOTF60% (r = 0.73, *p* < 0.05). Objective and subjective measures of DOF are not comparable due to differences in methodologies and criterions to define the level of degradation tolerance. Nevertheless, both objective and subjective measures showed a trend to a greater DOF for Tecnis Symfony and Tecnis Synergy IOLs compared to most of trifocal diffractive designs, with the exception of PanOptix.

## Introduction

Depth of focus is defined as “the perceptual tolerance of the eye to small retinal defocus”^1^. Its homologous in the object space is represented by the depth of field, that is the range of distances within a stimulus can be placed without obtaining a relevant degradation of the retinal image. Most of times both terms are used indistinctly as DOF. DOF is an optical phenomenon which is normally associated to the perception of blur since most of the measurement procedures of this variable depend on the subject response, although it can be also measured objectively^2^. Although DOF measures have been commonly relegated to experimental and research purposes, its clinical measurement is also of great value in cataract surgery as indicator of defocus tolerance with any type of intraocular lens (IOL) implanted. Conventional procedures to measure clinically the amplitude of accommodation can be used to measure the DOF when evaluating phakic subjects under cycloplegia^1^ or even in the case of pseudophakic subjects^3^ in which no residual accommodation is expected. As in the case of accommodation measures, one major problem with the measurement of DOF is the wide range of methodologies and criteria to establish what is optimal or not.

Subjective DOF can be obtained from the defocus curves, establishing a specific criterion of acceptable visual acuity (VA) reduction. This conventional procedure is well described^4^, being time consuming, but allowing to obtain in a simple way the maximum VA for each vergence with the use of trial lenses of different powers. However, this methodology is not exempt of inconveniences. Factors such as lenses step size^5^ or the order of presentation^6^ can be introduce some level of variability in the measurements, as well as optical factors as spectacle magnification and pupil reflex^7^. Alternatively, DOF can be also obtained by presenting a stimulus of a given size and asking the patient to define the range of distances for which the chart is perceived “equal” (same quality of vision). This methodology is highly dependent on what is defined as equal by each subject (detection of blur or changes on contrast)^8^, and this variability on subjects’ perception made some authors to adopt different criteria. Additionally, the size of the presented stimulus is another important factor to control when measuring DOF with this procedure since it has been demonstrated that stimulus size can affect the measures, and specifically higher DOF values are expected when using less restrictive criteria (increasing the size of the stimulus)^9^. The VA criteria to determine the DOF is critical for both methodologies mentioned above, being the restrictive criterion of 0.1 logMAR commonly used to define the defocus tolerance, although other criteria have been also defined^4,10–13^. The VA criterion to define the DOF can be absolute or relative^11^. Absolute criteria, based on the use of the same VA value to all subjects as a target, allow the comparison between individuals measured under the same conditions. On the other side, relative criteria seemed to be more convenient to characterize the real impact of defocus on patient’s vision since it considers the maximum VA of each subject and then apply the tolerance criteria.

On the other hand, objective DOF can be obtained from wavefront aberration data by defining the range of focus that provides a specific level of degradation on retinal image. Image quality meters (IQM) derived from aberrometry have demonstrated to correlate with patient visual performance^14^, and specifically a percentage of degradation on the Visual Strehl ratio based on the optical transfer function (VSOTF) has been proposed as an objective DOF measure that is good for clinical purposes^15,16^. Different threshold levels of degradation of the VSOTF (50%, 80% and 90%) have been proposed previously as a clinically useful measures of objective DOF^2,10,15,16^. Additionally, authors such as Yi et al.^16^ have analysed the relationship between subjective and objective measures of DOF, obtaining that a level of degradation of 60% on VSOTF could be the most representative of patients’ subjective perception^16^.

All these concepts have been used in several studies to analyze clinically the level of DOF in pseudophakic subjects implanted with different IOL designs^3,9,10,12,17,18^. The newest presbyopia correcting IOL designs are focused on providing significant levels of DOF, with even a continuous range of focus, allowing the patient to obtain a comfortable vision across a wide range of working distances. However, few studies provide a complete analysis of the level of DOF provided by these new designs of presbyopia-correcting IOLs using the different metrics available^10,19^. With this purpose, the aim of the present paper was to analyze the DOF obtained with different presbyopia correcting IOLs and to compare the results provided by each design. Additionally, different methodologies (defocus curves vs aberrometry) and criteria (absolute vs relative, different percentages of degradation on VSOTF) were also analyzed for each IOL design.

## Methods

### Patients

Participants were recruited from the Hospital Universitario Quirón (Madrid, Spain) and signed a written informed consent prior to the inclusion in the study according to the tenets of the Declaration of Helsinki. The protocol was approved by the Ethical Committee of Fundación Jimenez Díaz (CEIm-FJD) (Madrid, Spain). Participants who underwent cataract surgery with bilateral symmetrical IOL implantation were included. Subjects were implanted with one IOL or another depending on their clinical characteristics and the patient´s needs, and therefore no randomization was performed to assign one design or another to each group. Exclusion criteria were presence of pathological findings, history of systemic diseases and/or surgical complications during IOL implantation. No restrictions were applied regarding IOL lens power or patient´s refraction.

### Presbyopia-correcting IOLs

Patients were divided into 6 groups depending on the IOL implanted. Trifocal diffractive IOL designs used were AT LISA Tri (Carl Zeiss Meditec., Jena, Germany), FineVision (PhysIOL, Liège, Belgium), and PanOptix (Alcon Laboratories, Texas, USA). Furthermore, two extended depth of focus (EDOF) IOL’s were used, the Tecnis Symfony (Johnson & Johnson Vision, California, USA) and the MiniWell (SIFI MedTech, Catania, Italy). Tecnis Synergy IOL (Johnson & Johnson Vision, California, USA) which combines an EDOF and diffractive multifocal profile was also included in the evaluation.

### Subjective DOF

Defocus curves were obtained monocularly from all participants under photopic conditions at 3 months post-operatively. The step size in diopters was 0.50 D, ranging from + 3.00 to − 5.00 D. VA was measured in logMAR scale, and the optotype used was ETDRS (Precision Vision, Illinois, USA) at 4 m. All participants were measured with the best correction for far vision to compensate residual refractive errors. Subjective DOF was calculated using both absolute and relative criteria^5^. Absolute criterion was obtained from those vergences (in D) which provided VA values ≤ 0.1 logMAR. Relative criterion was obtained considering those vergences (in D) which provided a decay of 0.1 logMAR from to the best VA of each subject at zero vergence.

### Objective DOF

**Figure 1:**
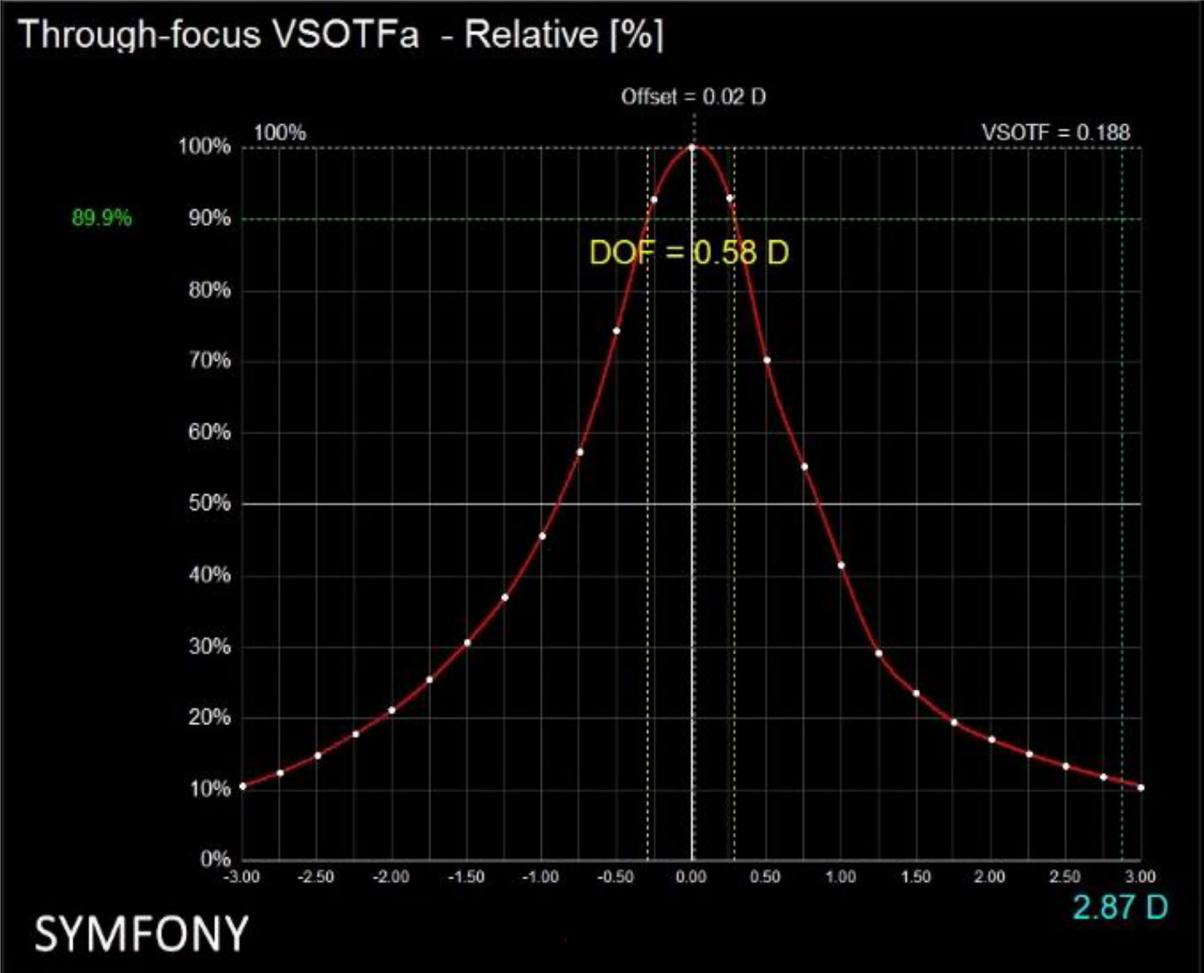
Report of DOF analysis in relative scale extracted from iTrace. The percentage of degradation on through-focus VSOTF is considered to calculate the DOF. In this case, a percentage of degradation of 90% was shown. Lower percentages represent a more permissive criterion of degradation, and will provide higher DOF results.

Aberrometry was measured with the iTrace system (Tracey Technologies, Texas, USA)^20^. This aberrometer provides some image quality metrics (IQM), and specifically the through-focus VSOTF for 90%, 80% and 60% threshold levels were considered to measure objective DOF as proposed previously^2,16,21^. VSOTF is the visual Strehl ratio of the optical transfer function and can be derived from the wavefront aberration data^2,16,21^. Aberrometry was performed under scotopic conditions with a scan size of 3.5 mm in all subjects, and none of the subjects had a pupil size under this value. Figure 1 shows an example of the report provided by the iTrace system for DOF.

### Statistical analysis

Statistical analysis of the results was done using the SPSS program v.19.0.0 for Windows (SPSS Inc., Chicago, IL). According to Kolmogorov–Smirnov test, some data were not normally distributed and then non-parametric tests were applied. One eye from each subject was randomly selected for analytical purposes to avoid biases from intrasubject correlation. Differences between groups were assessed with the Kruskal–Wallis test, and means were compared by pairs using the Mann–Whitney analysis with the Bonferroni adjustment. Comparison between subjective and objective DOF measurements was analyzed using the Wilcoxon test. Correlation between subjective and objective criteria was also assessed with the Spearman correlation coefficient. All statistical tests were 2-tailed, and p-values less than 0.05 were considered as statistically significant.

### Ethical approval

All procedures performed in studies involving human participants were in accordance with the ethical standards of the institutional research committee and with the 1964 Helsinki Declaration and its later amendments or comparable ethical standards.

### Informed consent

Informed consent was obtained from all individual participants included in the study.

## Results

Sample size was 150 eyes from 150 subjects (76 men and 74 women). Characteristics of the sample including mean age, pre-surgical spherical equivalent refraction, IOL power, and post-surgical spherical equivalent refraction are summarized per groups in Table 1. The level of statistical significance of differences between all IOL groups were analyzed, obtaining statistically significant differences in age (*p* < 0.001) and IOL power (*p* = 0.011) as well as in postoperative spherical equivalent (*p* < 0.001).

**Table 1:** Characterization in terms of age, preoperative and postoperative spherical equivalent (SE), and intraocular lens (IOL) power of the six IOL subgroups analyzed in the current study.

	Age (years)	Preop SE (D)	IOL Power (D)	Postop SE (D)
AT LISA TRI N = 25	60.68 ± 3.00 (56 to 65)	0.82 ± 1.15 (− 1.00 to 2.50)	20.48 ± 1.29 (18.00 to 22.00)	− 0.11 ± 0.23 (− 0.50 to 0.25)
FINEVISION N = 25	59.52 ± 2.35 (55 to 63)	0.74 ± 0.74 (− 0.75 to 2.00)	22.12 ± 1.68 (18.50 to 25.00)	− 0.26 ± 0.28 (− 0.75 to 0.25)
PANOPTIX N = 25	64.80 ± 2.68 (61 to 70)	0.12 ± 1.32 (− 1.50 to 3.00)	21.66 ± 1.86 (17.00 to 26.00)	− 0.13 ± 0.21 (− 0.50 to 0.25)
SYMFONY N = 25	73.88 ± 4.46 (68 to 83)	0.77 ± 1.39 (− 1.75 to 3.25)	21.24 ± 2.31 (16.00 to 25.50)	0.25 ± 0.28 (− 0.25 to 0.75)
SYNERGY N = 25	66.16 ± 5.11 (55 to 74)	0.17 ± 1.07 (− 1.50 to 2.75)	21.86 ± 2.53 (15.50 to 28.50)	0.10 ± 0.35 (− 0.50 to 0.75)
MINI WELL N = 25	61.96 ± 2.17 (59 to 68)	0.94 ± 1.24 (− 2.00 to 3.75)	21.64 ± 2.13 (14.50 to 25.50)	− 0.19 ± 0.33 (− 0.75 to 0.50)
Kruskal–Wallis p− value	< 0.001	0.053	0.011	< 0.001

**Figure 2:**
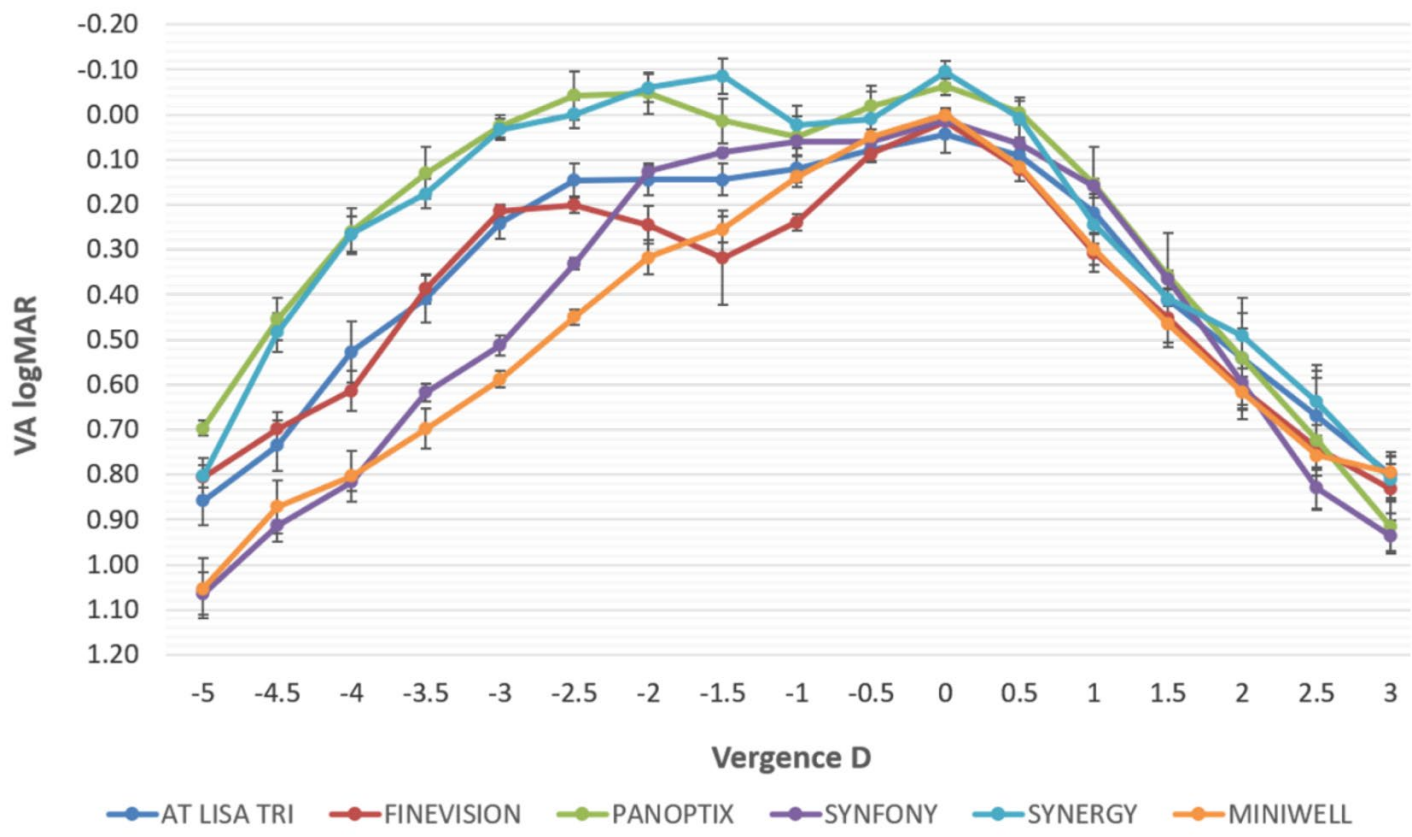
Mean defocus curves obtained in each intraocular lens (IOL) group.

Figure 2 shows the mean defocus curves obtained in IOL groups from which subjective DOF measures were calculated. Table 2 summarizes the post-operative visual acuity values obtained from defocus curves for each group for far (vergence + 0.00D), intermediate (vergence − 1.00D) and near vision (vergence −2.50D). For far vision, all IOLs showed statistically significant differences with some of the other designs evaluated (*p* < 0.001), with the exception of AT LISA tri vs Finevision (p 0.195), AT LISA tri vs Symfony (p 0.195), Finevision vs Symfony (*p* > 0.999), Finevision vs Miniwell (*p* > 0.999), and Symfony vs Miniwell (p 0.405). DOF was measured under both subjective and objective conditions, being these results also summarized in Table 3. Differences were also analyzed by pairs, and comparisons between groups revealed that there were statistically significant differences between some IOL groups. Specifically, subjective DOF measures using the absolute criterion differed significantly for all comparisons between pairs (*p* < 0.001), except for the comparisons AT LISA tri vs. MiniWell (*p* = 0.060) and FineVision vs. MiniWell (*p* = 0.999). In contrast, no significant differences in subjective DOF measures using the relative criterion were found for the comparisons between AT LISA tri vs. PanOptix (*p* = 0.999), AT LISA tri vs. Tecnis Symfony (*p* = 0.999), PanOptix vs. Tecnis Symfony (*p* = 0.999), FineVision vs. Tecnis Synergy (*p* = 0.999), FineVision vs. MiniWell (*p* = 0.999), Tecnis Synergy vs. MiniWell (*p* = 0.999) and Tecnis Symfony vs. Tecnis Synergy (*p* = 0.070).

**Table 2:** Summary of post-operative best-corrected visual acuity obtained by defocus curves for far, intermediate and near distance of the six IOL subgroups analyzed in the current study.

	DCVA (+ 0.00 D)	DCIVA (− 1.00 D)	DCNVA (− 2.50 D)
AT LISA TRI N = 25	0.04 ± 0.04 (− 0.02 to 0.12)	0.12 ± 0.03 (0.02 to 0.18)	0.15 ± 0.04 (0.04 to 0.24)
FINEVISION N = 25	0.02 ± 0.03 (− 0.02 to 0.06)	0.24 ± 0.02 (0.22 to 0.28)	0.20 ± 0.02 (0.16 to 0.24)
PANOPTIX N = 25	− 0.06 ± 0.02 (− 0.08 to − 0.04)	0.05 ± 0.04 (0.02 to 0.12)	− 0.04 ± 0.05 (− 0.14 to 0.00)
SYMFONY N = 25	0.01 ± 0.02 (− 0.04 to 0.06)	0.06 ± 0.02 (0.04 to 0.08)	0.33 ± 0.01 (0.32 to 0.36)
SYNERGY N = 25	− 0.09 ± 0.02 (− 0.16 to − 0.02)	0.02 ± 0.04 (− 0.02 to 0.14)	0.00 ± 0.03 (− 0.08 to 0.06)
MINI WELL N = 25	0.00 ± 0.01 (− 0.02 to 0.02)	0.14 ± 0.02 (0.10 to 0.18)	0.45 ± 0.02 (0.40 to 0.48)
Kruskal–Wallis p-value	*p* < 0.001	*p* < 0.001	*p* < 0.001

Visual acuity was expressed in logMAR scale. DCVA: distance corrected visual acuity for vergence zero; DCIVA: distance corrected intermediate visual acuity for vergence − 1.00 D; DCNVA: distance corrected near visual acuity for vergence − 2.50 D.

Regarding objective DOF measures for the 90% threshold level, no statistically significant differences were found for the following comparisons: AT LISA tri vs. FineVision (*p* = 0.999), AT LISA tri vs. MiniWell (*p* = 0.075), FineVision vs. MiniWell (*p* = 0.075), PanOptix vs. Tecnis Synergy (*p* = 0.999), and Tecnis Symfony vs. Tecnis Synergy (*p* = 0.150). Regarding objective DOF measures for the 80% threshold level, no statistically significant differences were found for the following comparisons: AT LISA tri vs. FineVision (*p* = 0.090), PanOptix vs. Tecnis Symfony (*p* = 0.060), PanOptix vs. Tecnis Synergy (*p* = 0.999), and Tecnis Symfony vs. Tecnis Synergy (*p* = 0.075). Concerning objective DOF measures for the 60% threshold level, statistically significant differences were found for all comparisons with the exemption of PanOptix vs. Tecnis Synergy (*p* = 0.999).

Comparison between subjective and objective DOF showed that subjective measures were significantly higher in all IOL groups (*p* < 0.001) (Table 3). Concerning the correlation analysis by groups, no significant correlations were found between subjective (absolute and relative) and objective criteria (VSOTF90, 80 and 60) for any group with the exception of: relative vs VSOTF80 (0.49, p 0.01) in Panoptix group, and relative vs VSOTF80 (0.49 p 0.01) in Synergy group.

**Table 3:** Summary of the depth of field (DOF) measurements obtained under both subjective and objective conditions.

	Absolute Criteria VA ≤ 0.1 logMAR	Relative Criteria VA max + 0.1 logMAR	VSOTF 90%	VSOTF 80%	VSOTF 60%
AT LISA TRI N = 25	1.60 ± 0.66 (0.50 to 4.00)	2.60 ± 1.01 (1.50 to 4.00)	0.31 ± 0.04 (0.23 to 0.39)	0.51 ± 0.03 (0.48 to 0.57)	0.72 ± 0.03 (0.68 to 0.79)
FINEVISION N = 25	1.10 ± 0.32 (0.50 to 1.50)	1.30 ± 0.25 (1.00 to 1.50)	0.30 ± 0.04 (0.24 to 0.42)	0.54 ± 0.04 (0.49 to 0.60)	0.76 ± 0.03 (0.70 to 0.81)
PANOPTIX N = 25	3.84 ± 1.21 (1.50 to 4.50)	2.88 ± 1.20 (1.50 to 4.00)	0.43 ± 0.06 (0.31 to 0.52)	0.83 ± 0.04 (0.78 to 0.89)	1.56 ± 0.05 (1.48 to 1.63)
SYMFONY N = 25	2.56 ± 0.17 (2.50 to 3.00)	2.70 ± 0.46 (1.50 to 3.50)	0.50 ± 0.07 (0.40 to 0.62)	0.80 ± 0.05 (0.70 to 0.91)	1.17 ± 0.04 (1.10 to 1.25)
SYNERGY N = 25	3.90 ± 0.52 (1.50 to 4.50)	1.66 ± 1.21 (0.50 to 3.50)	0.45 ± 0.05 (0.38 to 0.55)	0.83 ± 0.03 (0.78 to 0.90)	1.53 ± 0.04 (1.47 to 1.60)
MINI WELL N = 25	1.22 ± 0.33 (1.00 to 2.00)	1.26 ± 0.36 (1.00 to 2.00)	0.34 ± 0.04 (0.28 to 0.41)	0.71 ± 0.03 (0.65 to 0.77)	1.01 ± 0.05 (0.95 to 1.12)
Kruskal–Wallis p-value	< 0.001	< 0.001	< 0.001	< 0.001	< 0.001

## Discussion

### Absolute versus relative subjective DOF criteria

Subjective DOF obtained by defocus curves has been previously studied by some authors in eyes implanted with monofocal^3,11–13,22^, bifocal^10–13,22^ and trifocal IOLs^3,10^, but the variability on the used criteria among studies complicates significantly the comparison between them^1^. Nevertheless, very similar DOF values were found using the relative criterion in eyes implanted with the trifocal IOL AT LISA tri (⁓2.50 D), when comparing our results with those obtained by Barisic et al.^3^. Likewise, some of the studied IOL designs in the current study were also analysed in a previous study of our research group^10^ (AT LISA tri, FineVision, Tecnis Symfony and MiniWell), obtaining clinically significant higher values of DOF in the present paper although the same procedures and criteria were used. These differences may be explained by the neuroadaptation^23^, since in the current study the results were analysed at 3 months postoperatively (vs. one month postoperatively in the previous paper), allowing the visual system to adapt to the retinal defocus induced with these IOL designs. Another explanation to these differences between studies may be the use of steps of 0.5 D when measuring the defocus curve compared to the steps of 0.25 D used in the previous series ^5^, which implies the assumptions of some errors when defining the subjective DOF and considering VA values smaller or equal to 0.1 logMAR.

Regarding the use of the absolute criterion, those IOL designs providing better VA values for far vision showed significantly higher DOF values (PanOptix and Tecnis Synergy). Absolute criteria considered the limit in 0.1 logMAR for all subjects, and then it is expected that those IOLs in which subjects achieved better VA also needed more changes in the defocus curves lenses to reach this degradation, but the form in which varies the curve will also affect the obtained results. In the present study, PanOptix, Tecnis Synergy and Tecnis Symfony showed the higher absolute DOF values, coinciding with those designs providing the best VA for far vision (see Fig. 2). The analysis of the relative criterion considers these differences on far vision VA and allows the comparison of the level of visual degradation across distances (if VA decreases more or less rapidly with the distance). In the current study, the PanOptix, AT LISA tri and Tecnis Symfony groups showed a more attenuated decrease of VA with the change in distance, therefore providing better DOF results with this relative criterion. As shown, different results can be obtained according to the criterion used for defining the subjective DOF, being from our perspective the analysis of both relative and absolute criteria necessary to obtain a complete overview of the any presbyopia-correcting IOL performance. Buckhurst et al.^11^ analysed the differential aspects between multifocal designs using both the absolute and relative criteria, concluding that the relative criterion is not good for comparing the DOF between IOLs since no differences were found when comparing multifocal and monofocal designs. In our sample, the relative criterion also provided some confusing results, as happened with the Synergy IOL. This IOL showed a relatively flat defocus curve around 0.00 logMAR, a mean DOF of 3.90 ± 0.52 D using the absolute criterion, one of the largest DOF values measured objectively according to VSOTF60%, but a mean DOF of 1.66 ± 1.21 D using the relative criterion. For this reason, the subjective DOF values obtained with the relative criterion should be considered with caution and compared to those obtained subjectively using the absolute criterion and to those obtained objectively.

Some authors have also proposed an alternative methodology for a better calculation of DOF, the area-of-metric method^11^, demonstrating to be more sensitive to multifocal IOL differentiation by using defocus curves. This proposed methodology consisted of adjusting a polynomial regression curve fitting the results obtained experimentally and calculating the DOF mathematically using the Newton–Raphson method to obtain those points of the curve which met an established criterion. DOF was then calculated as the dioptric distance over which VA was better than 0.3 logMAR. This methodology, although provide more exact calculations on DOF results for research purposes, is time consuming on clinical practice and require mathematical expertise that may not be adequate for practitioners on clinical routine. The main difficulty that was found on trying to apply this method in the current study was to find a curve that really fitted our experimental results exactly enough to perform afterwards predictions and calculations. For this reason, only absolute and relative conventional criteria were applied. 

### Percentage of degradation on VSOTF

Image Quality Metrics (IQMs) derived from wavefront aberration data have demonstrated to be good predictors of visual performance^14^ and specifically VSOTF have been proposed by some authors as one of the best parameters to quantify objectively the DOF^16,21^. As in the case of subjective measures, a criterion must be established to determine the level of degradation which is acceptable, and this criterion will affect the obtained results and therefore the DOF value. Different methodologies and levels of degradation (50%, 60%, 80% and 90%) have been proposed previously by many authors^2,16,21^ to determine the DOF. In the present study, the different presbyopia-correcting IOL designs were compared using three different criteria (60%, 80% and 90%) to objectively determine the visual performance.

Results from the present study revealed that the DOF achievable with the IOL designs studied did not totally depend on the type of IOL, trifocal, EDOF or Tecnis Synergy, since individual differences were found between IOLs. Although the EDOF IOLs are supposed to provide better DOF due to its continuous range of focus, as demonstrated in a previous work^10^, the Tecnis Synergy IOL showed results comparable to those provided by the EDOF IOL (Tecnis Symfony) and the trifocal diffractive IOL PanOptix, and even better than MiniWell for all studied criteria defined.

In contrast to subjective measures that depend on individual subjects perception and neuroadaptation^8^, the comparison of the objective DOF results considering the 90% of VSOTF from the current study with those results obtained in the previous study from our research group^10^, results showed that DOF did not differed remarkably in general terms. Similar DOF values in both studies were found for the FineVision (0.29 vs 0.30 D), AT LISA tri (0.36 vs 0.31 D), Tecnis Symfony (0.50 vs 0.50 D) and MiniWell (0.36 vs 0.34 D) IOLs. Regarding the analysis of DOF using the levels of degradation of 80% and 60%, Tecnis Synergy, Tecnis Symfony and PanOptix showed the best results. As expected according to previous researches^9^, a more permissive criterion provide a higher tolerance and therefore a longer DOF for all IOLs.

### Comparison between subjective and objective DOF

Several authors have studied in the past the differences between subjective and objective DOF measures^2,10,16,21^, demonstrating that subjective ones’ overestimate results obtained objectively. The evaluation of the whole visual performance, including the neural function^21^ in comparison to only the optical part, and the subjective tolerance to blur^8,24^ may allow some subjects to detect some optotypes in spite of not seeing them clearly, leading to a potentially better performance compared to the objective criterion of the IQMs. This would explain differences between objective and subjective measures of DOF, although some other factors have to be also considered ^1^. Pupil size is another parameter that should be assessed since it has been demonstrated that it affects DOF results in pseudophakic subjects^3^, and could explain the higher DOF results obtained in the subjective measurement under photopic conditions against the scotopic objective measurement. Pupillometry of each individual on each measure was not evaluated in the present paper so this hypothesis could not be tested.

Regarding the correlation between subjective and objective measures for the whole sample, the higher correlation was found between the absolute criteria and VSOTF60. These results are according to those obtained by Yi et al.^16^ who studied the relationship between subjective and objective measures through two threshold levels (50% and 80%), concluding that DOF values associated to 60% of VSOTF were the most representative of subjective measures. These results cannot be directly compared to the results of the present study, since DOF was measured based on just noticeable blur and Badal stage movement. Additionally, in our case, no more degradation levels behind 60% were studied, so we cannot affirm that this level is the most representative, but it seems that higher levels are not appropriate to be compared with patient´s subjective report.

On the other hand, results from the whole sample analysis showed weak or no correlation of the subjective relative criteria with objective VSOTF criteria. Relative criteria are applied from the maximum visual acuity of each subject, and therefore those designs providing better VA, will not benefit by this criterion as occurs with the absolute criteria. In the case of objective DOF, VSOTF has proved to be a good descriptor of visual performance and has been correlated to VA^14^, that is the more altitude of the peak in Fig. 1 the better performance, but in terms of DOF it does not mean higher results.

The faster or slower degradation that exists within designs depending on the optical profile of the IOL, will also affect the DOF results and may be the cause of the differences found when analysing the correlation of DOF criteria (subjective vs objective) by groups of presbyopia-correcting IOLs. Different levels of correlation between objective and subjective DOF are expected among IOL designs depending on the maximum peak and rate of degradation. Indeed, our analysis by groups revealed that correlation between subjective relative criteria and the objective DOF was present only for these designs providing better VA (Panoptixand Synergy). Some other factors such as difference in pupil size and corneal aberrations between groups may have contributed to these differences. More studies are needed to confirm these results in terms of differences between subjective and objective DOF as a function of the optical profile of the IOL using larger sample sizes.

## Conclusion

The Tecnis Symfony and Tecnis Synergy IOLs seemed to provide a wide range of DOF for both subjective and objective measures, tending to be superior to most of the trifocal designs except for PanOptix. The use of different criteria to define the tolerance to defocus, absolute or relative with subjective measures and the level of degradation accepted on VSOTF with objective measures, provide different DOF values that cannot be directly compared.
